# TS-1 add-on therapy in Japanese patients with triple-negative breast cancer after neoadjuvant or adjuvant chemotherapy: a feasibility study

**DOI:** 10.1007/s10637-019-00829-w

**Published:** 2019-07-10

**Authors:** Kenichi Inoue, Shigenori E. Nagai, Tsuyoshi Saito, Takashi Sakurai, Kei Kimizuka, Hirofumi Yamada, Toru Kuroda, Satoshi Hata, Yasuo Yamazaki, Masato Kojima, Kazushige Futsuhara, for the SBCCSG-14 investigators

**Affiliations:** 1 Division of Breast Oncology, Saitama Cancer Center, 780 Komuro, Ina-machi, Kita-adachi-gun, Saitama, 362-0806 Japan; 2 Department of Breast Surgery, Saitama Red Cross Hospital, Saitama, 330-8553 Japan; 3 Division of Breast Surgery, JCHO Saitama Medical Center, Saitama, 330-0074 Japan; 4 Department of Breast Surgery, Kasukabe Medical Center, Saitama, 344-8588 Japan; 5 Department of Surgery, Sekishindo Hospital, Saitama, 350-1123 Japan; 6 Breast Center, Mitsui Hospital, Saitama, 350-0066 Japan; 7 Division of Surgery, Ina Hospital, Saitama, 362-0806 Japan; 8 Department of Breast, Dokkyo Medical University, Saitama Medical Center, Saitama, 343-8555 Japan; 9 Department of Surgery, Saitama Medical Center, Jichi Medical University, Saitama, 330-8503 Japan

**Keywords:** TS-1, Add-on therapy, Triple-negative breast cancer, Neoadjuvant chemotherapy, Adjuvant chemotherapy, Feasibility

## Abstract

*Purpose* We examined the feasibility, efficacy, and safety of TS-1 add-on therapy (TAT) in Japanese patients with triple-negative breast caner (TNBC). *Methods* TAT (TS-1, 80 mg/m^2^/day, BID, PO), consisting of the 21-day cycles of 14-day consecutive administration followed by 7-day drug holiday, was conducted for 365 days. The median follow-up was 75.2 months (range, 7.3–103.3 months). The primary endpoint was the feasibility of TAT. The secondary endpoints included relapse-free survival (RFS), overall survival (OS), and safety. *Results* 63 Japanese patients with TNBC (median age, 52.5 years; range, 23.7–68.6 years) were examined. Among them, 34 (54.0%) were postmenopausal, 54 (93.7%) had TNBC of common histological type, 51 (81.0%) had T1 to 3 tumors, 63 (100%) had undergone standardized surgery, and 44 (69.8%) and 19 (30.2%) had undergone neoadjuvant chemotherapy and adjuvant chemotherapy, respectively. The 365-day cumulative rate of TS-1 administration was 68.3% (95% confidence interval, 55.3–79.4), being comparable to 65.8% previously reported for gastric cancer. The 5-year RFS rates were 52.3% and 84.2% in the neoadjuvant and adjuvant chemotherapy groups, respectively, and the 5-year OS rates were 68.0% and 89.5%, respectively. The most common adverse events (AEs) were leucocyte count decreased (50.8%), total bilirubin decreased (44.4%), and pigmentation (42.9%). AEs were manageable clinically, and any grade 4 AEs did not develop. *Conclusions* The 365-day cumulative rate of TS-1 administration in TNBC patients was comparable to that in gastric cancer patients despite previous chemotherapy with anthracyclines and/or taxanes. TAT was feasible for TNBC patients after standard primary therapy.

## Introduction

Adjuvant systemic therapies including chemotherapy and endocrine therapy after surgery have reduced the relapse rate to approximately two-thirds in patients with breast cancer involving micrometastases [1–3]. Many of patients who failed to gain pathologic complete response (pCR) by neoadjuvant chemotherapy present the postoperative relapse of residual invasive tumor. Therefore, further improvements in treatment outcomes are expected. Currently, patients with estrogen receptor (ER)-, progesterone receptor (PgR)-, and human epidermal growth factor receptor 2 (HER2)-negative—triple-negative—breast cancer (TNBC) are treated by standard primary therapy (SPT) consisting of standardized surgery and neoadjuvant/adjuvant chemotherapy, or radiotherapy as needed; however, TNBC may relapse in as promptly as 1 year after adjuvant/neoadjuvant chemotherapy and post-relapse prognosis is very poor. Posttreatment for TNBC patients who underwent standardized surgery and/or adjuvant/neoadjuvant chemotherapy is not yet established. Therefore, there are clinical needs to develop a new posttreatment for them and to investigate its feasibility.

TS-1, an oral fluoropyrimidine anticancer agent, is a combination formulation that contains tegafur (a prodrug of 5-fluorouracil [5-FU]) and two biochemical modulators—gimeracil (a competitive inhibitor of dihydropyrimidine dehydrogenase that is involved in 5-FU degradation, resulting in the enhancement of the antitumor effect of 5-FU) and oteracil potassium (a competitive inhibitor of orotate phosphorybosyltransferase that is widely distributed in the gastrointestinal tract, reducing the gastrointestinal toxicities of 5-FU)—at a molar ratio of 1:0.4:1 [4–6]. In Japan, TS-1 has been approved to treat a wide array of solid malignancies (gastric cancer, colorectal cancer, head and neck cancer, non-small-cell lung cancer, inoperable or relapsed breast cancer, pancreatic cancer, and biliary cancer) [7–14].

The objective of the present study was to exploratively examine the feasibility, efficacy, and safety of TS-1 adjuvant chemotherapy added to SPT—TS-1 add-on therapy (TAT)—in Japanese patients with TNBC who were at high risk for relapse despite having undergone SPT.

## Materials and methods

### Study design

This was a multicenter, open-label, feasibility study of TAT and included patients who were at post-adjuvant chemotherapy high risk for relapse due to residual invasive tumor or lymphnode metastases that developed despite having undergone neoadjuvant chemotherapy. The pathological response grades to neoadjuvant chemotherapy were assessed according to the histopathological criteria provided by the Japanese Breast Cancer Society [15]. The criteria defines pathological complete response (pCR) as the total disappearance of infiltrates, including lymph node infiltrates, regardless of the presence of residual ductal carcinoma in situ.

### Study population

Between July 2008 and September 2012, a total of 63 Japanese patients with TNBC were enrolled at 9 medical institutions in Japan. Patients were considered eligible when meeting all of the following criteria at the time of enrollment: histopathologically confirmed breast cancer; negativity for all of ER, PgR, and HER2; conduction of primary therapy; 20 to 75 years of age; 0 to 1 in Eastern Cooperative Oncology Group (ECOG) performance status; capability of oral intake; conserved functionality of major organs; and provision of written informed consent to study enrollment by the patient herself. Primary therapy consisted of standardized surgery and neoadjuvant/adjuvant chemotherapy. Patients had residual invasive tumor after neoadjuvant chemotherapy or underwent adjuvant chemotherapy. Radiotherapy was conducted on an as needed basis. Standardized surgery was defined as mastectomy, partial resection, sentinel lymph node biopsy, or lymph node dissection. Chemotherapy was defined as neoadjuvant or adjuvant chemotherapy with anthracyclines (doxorubicin and epirubicin) or taxanes (docetaxel and paclitaxel). The exclusion criteria included the following reasons: surgical samples that were positive for either of ER, PgR, or HER2; male patients with breast cancer; serious complications; a history of serious allergy to fluoropyrimidine-derived antineoplastic agents; pregnancy or suspected pregnancy; and inadequacy to study enrollment as adjudged by the attending physician. All patients provided written informed consent at the time of enrollment. The study protocol was approved by the Institutional Review Board or Central Ethics Committee of the participating medical institutions. Patients were followed up at the regular visits to the hospital during 5 years to check for relapse after TAT. The present study was registered (University Hospital Medical Information Network identifier: 000001414).

### Rationales for the determination of the dose and dosing schedule

Kinoshita et al. [16] conducted a feasibility study of adjuvant chemotherapy with TS-1 (80–120 mg/body per day) for 1 year in patients with curatively resected gastric cancer and concluded that the therapy was feasible as adjuvant chemotherapy for gastric cancer. The Adjuvant Chemotherapy Trial of TS-1 for Gastric Cancer (ACTS-GC) Group (2007) [9] reported in 1,059 patients with stage II or III A/B gastric cancer who had undergone gastrectomy with extended lymph node dissection, that the TS-1 group produced 32% and 38% reductions in death and relapse risk, respectively, as compared with the surgery-only group. Based on the dose found to be effective, therefore, we employed the dose of 80 mg/m^2^/day, BID, PO, to examine the tolerability of TS-1 for 1 year. Moreover, Tsukuda et al. [17] conducted a feasibility study of the randomized scheduling of TS-1 in 101 patients with advanced head and neck cancer to examine the following two dosing schedules lasting 24 weeks: 1) 4-week consecutive administration followed by 2-week drug holiday and 2) 2-week consecutive administration followed by 1-week drug holiday. In the latter dosing schedule group, they found the treatment effect comparable to that in the former group, the good completion rate and cumulative total dose, the OS rate sufficient for 365-day administration, as well as the significantly different incidences of diarrhea and dermatological symptoms. Since all these efficacy and safety profiles of TS-1 permitted us to expect a high treatment effect, a high drug compliance rate, and less toxicities, we decided to employ the dose of TS-1 80 mg/m^2^/day, BID, PO, and the dosing schedule of 14-day consecutive oral administration followed by 7-day drug holiday. Therefore, dose or dosing schedule modification as occurred in the previous feasibility studies of TS-1 was not required to find the optimal dose or dosing schedule of TS-1.

### Administration

TS-1 (80 mg/m^2^/day, BID, PO) was administered according to the dosing schedule consisting of the 21-day cycles of 14-day consecutive oral administration followed by 7-day drug holiday. This regimen was repeated for the scheduled 365-day treatment period unless relapse occurred.

### Primary endpoint, secondary endpoints, and exposure to TS-1

The primary endpoint was the feasibility, efficacy, and safety of TAT. The feasibility was defined as the successful conduct of TAT for 365 days based on the completed schedules of TAT as planned, as well as on the dose reductions, delays, and interruptions of TS-1. The secondary endpoints were relapse-free survival (RFS), relative dose intensity (RDI), OS, and safety. RFS was defined as the period between study enrollment and the occurrence of an event (relapse or death) whichever came first. RDI, defined as the proportion of the actual dose to the scheduled dose, was calculated for TS-1. The actual dose was determined based on the drug compliance notebook in which the patient was instructed to note the number of taken capsules. RDI, as well as the rates of dose reduction, delay, and interruption were calculated to assess the sustainability of TAT by means of exposure to TS-1 during the study. OS was defined as the period between study enrollment and death. All deaths were considered as events regardless of their causality with TAT.

### Safety

Adverse events (AEs) were defined as all undesired or unintended signs, symptoms, or disorders that developed to patients who received TS-1, and no regard was given to their causality with TS-1. All changes, which were abnormal compared with the baseline values prior to cycle 1, were considered as AEs that were graded according to the Japanese version of the National Cancer Institute Common Terminology Criteria for Adverse Events (CTCAE) version 3.0 (National Cancer Institute 2007) [18].

### Statistical analyses

The full analysis set analyses were made for the primary and secondary endpoints. Kaplan-Meier estimates were used for the 365-day cumulative rates of TS-1 administration, RFS, and OS. The required number of patients was calculated using the optimal two-stage design model proposed by Englert et al. [19] according to the following conditions: the expected 365-day cumulative rate of TS-1 administration, 60%; the threshold for the cumulative rate, 40%; α = 0.1; and β = 0.1. The required number of patients was calculated to be 54. However, the target number of patients was set to be 60 in consideration of possible ineligible patients. SPSS version 19 (IBM, Armonk, NY) was used to make all statistical analyses. Follow-up was complete and closed in July 2017, 5 years after the last patient was enrolled in the study.

## Results

### Study population

Clinicopathological characteristics of patients at baseline are shown in Table 1. A total of 63 Japanese patients with TNBC (median age, 52.5 years; range, 23.7–68.6 years) were enrolled. Among them, 34 (54.0%) were postmenopausal, 54 (93.7%) had TNBC of common histological type, 51 (81.0%) had T1–3 primary tumors, 63 (100%) had undergone standardized surgery, and 44 (69.8%) and 19 (30.2%) had undergone neoadjuvant chemotherapy and adjuvant chemotherapy, respectively. In addition, 97.7% of patients had undergone neoadjuvant chemotherapy with an anthracycline and a taxane (i.e., 63.6% and 34.1% of them had undergone sequential therapy and concurrent therapy, respectively). Thirty-nine patients (88.7%) in the neoadjuvant chemotherapy group had breast cancer of pathological effect grades 1 to 3. All patients underwent standardized surgery, and 97.7% of patients who underwent neoadjuvant therapy and 63.2% of patients who underwent adjuvant therapy received anthracyclines and taxanes. The median follow-up was 75.2 months (range, 7.3–103.3 months).

**Table 1:** Clinicopathological characteristics of patients at baseline

	Study groups (*N* = 63)	
Characteristics	Neoadjuvant group (*n* = 44)	Adjuvant group (*n* = 19)
Age at baseline, yrs		
Median	51.8	53.9
Range	23.7–68.6	35.6–68.2
Menopausal status, n (%)		
Premenopausal	22 (50.0)	7 (36.8)
Postmenopausal	22(50.0)	12(63.2)
Histology, n (%)		
Common type	40(90.9)	19(100.0)
Others	4(9.1)	0(0)
T - primary tumor, n (%)		
T1	0(0)	5(26.3)
T2	23(52.3)	11(57.9)
T3	9(20.5)	3(15.8)
T4	12(27.3)	0(0)
N - regional lymph nodes, n (%)		
N0	7(15.9)	12(63.2)
N1	21(47.7)	7(36.8)
N2	7(15.9)	0(0)
N3	9(20.5)	0(0)
Pathological response grades, n (%)*		
0	3(6.8)	Not applicable
1a or 1b	20(45.5)	Not applicable
2 or 3	19(43.2)	Not applicable
Not evaluable	2(4.5)	Not applicable
Number of lymph nodes involved in histological assessment, n (%)		
0	19(43.2)	9(47.4)
1–3	14(31.8)	4(21.1)
≥4	11(25.0)	5(26.3)
Surgery, n (%)		
Mastectomy	14(31.8)	9(47.4)
Partial resection	30(68.2)	10(52.6)
Neoadjuvant or adjuvant chemotherapy, n (%)		
Sequential anthracyclines and taxanes	28(63.6)	12(63.2)
Concurrent anthracyclines and taxanes	15(34.1)	0(0)
Anthracycline-containing chemotherapy alone	1(2.3)	6(31.6)
Taxane-containing chemotherapy alone	0(0)	1(5.3)
Fluorouracil plus anthracyclines	10(22.7)	5(26.3)
Radiotherapy, n (%)		
Present	40(90.9)	12(63.2)
Absent	4(9.1)	7(36.8)

*The pathological responses to neoadjuvant chemotherapy were graded from 0 to 3 according to the histopathological criteria for assessment of therapeutic response provided by the Japanese Breast Cancer Society [Kurosumi et al. Breast Cancer 2001;8(1):1–2]. Grade 0 (no response): Almost no change in cancer cells after treatment. Grade 1 (slight response). Grade 1a (mild response): Mild changes in cancer cell regardless of the area, or marked changes in cancer cell seen in less than one-third of cancer cells. Grade 1b (moderate response): Marked changes in one third or more but less two-thirds of tumor cells. Grade 2 (marked response): Marked changes in two thirds or more of tumor cells. Grade 3 (complete response): Necrosis or disappearance of all tumor cells. Replacement of all cancer cells by granuloma-like and/or fibrous tissue. In the case of complete disappearance of cancer cells, pretreatment pathological evidence of the presence of cancer is necessary

### Cumulative rate of TS-1 administration, RDI, RFS, and OS

**Fig. 1:**
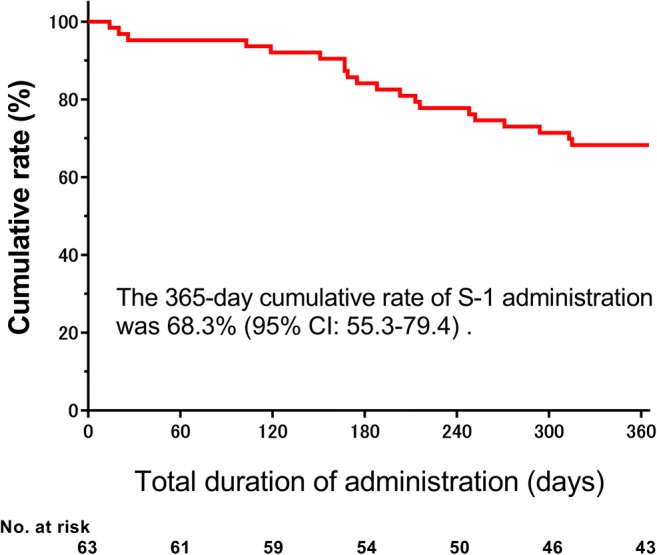
365-day cumulative rates of TS-1 administration. CI, confidence interval

**Fig. 2:**
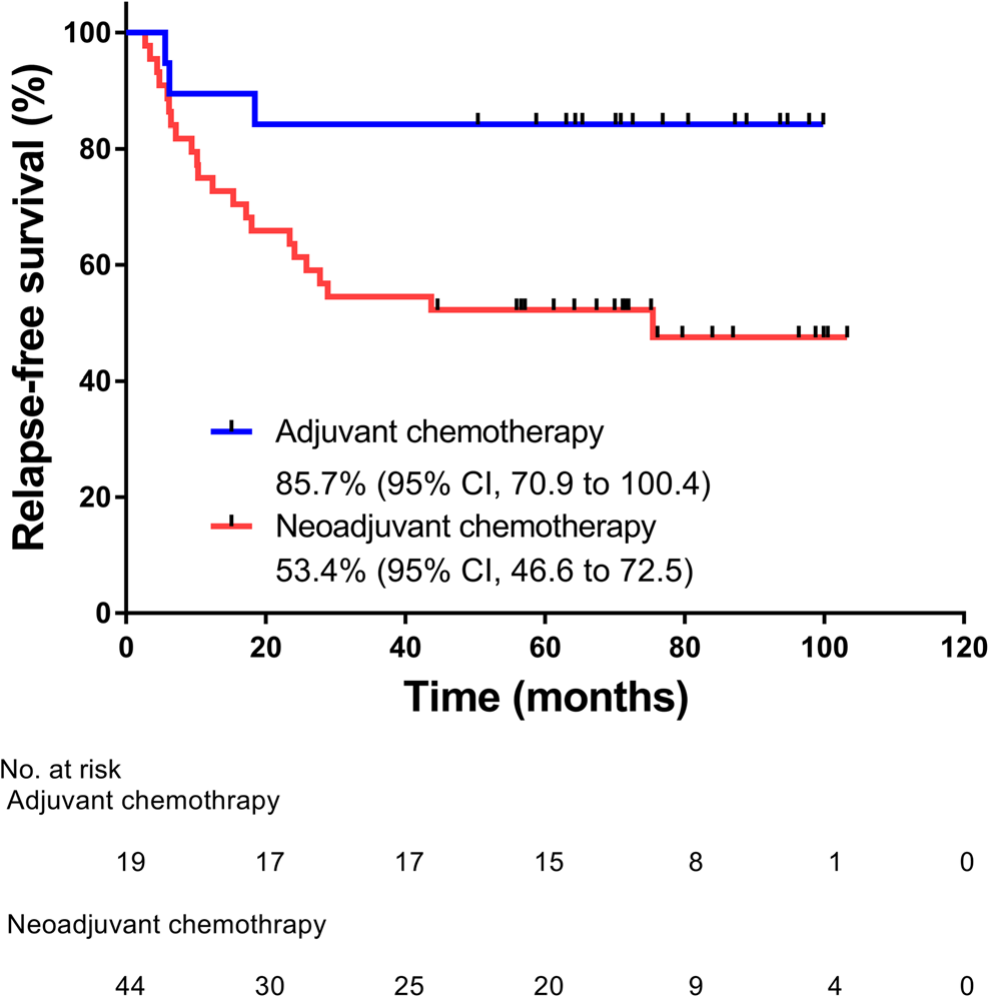
Rates of relapse-free survival in the neoadjuvant and adjuvant chemotherapy groups. CI, confidence interval

**Fig. 3:**
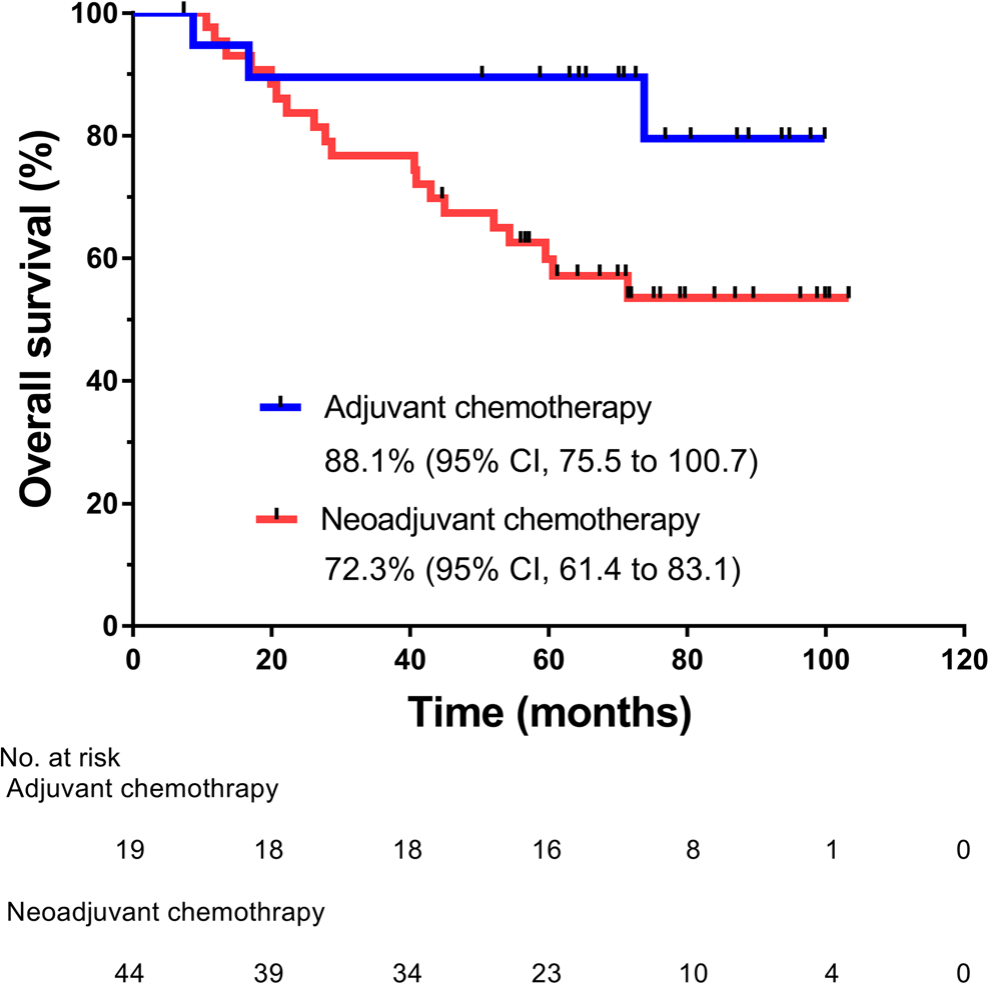
Rates of overall survival in the neoadjuvant and adjuvant chemotherapy groups. CI, confidence interval

The 365-day cumulative rate of TS-1 administration was 68.3% (95% CI, 55.3–79.4) (Fig. 1), and the mean RDI was 72.3% in patients who underwent 18 cycles (Table 2). Fifteen patients (34.1%) in the neoadjuvant chemotherapy group and 8 patients (42.1%) in the adjuvant chemotherapy group underwent the cycles and doses as scheduled (Table 2). These results indicate that TS-1 was well tolerated by most patients who had been treated by neoadjuvant/adjuvant chemotherapy. In the neoadjuvant chemotherapy group including patients who had a non-pCR, the mean RFS rate was 53.4% (95% CI, 46.6 to 72.5) (Fig. 2); the 3- and 5-year RFS rates were 53.4% and 52.3%, respectively. In the adjuvant chemotherapy group including patients who underwent postoperative chemotherapy with anthracyclines or taxanes, the mean RFS rate was 85.7% (95% CI, 70.9 to 100.4) (Fig. 2); the 3- and 5-year RFS rates were 84.2% each. The mean OS rate was 72.3% (95% CI, 61.4 to 83.1) in the neoadjuvant chemotherapy group and was 88.1% (95% CI, 75.5 to 100.7) in the adjuvant chemotherapy group (Fig. 3). The 3- and 5-year OS rates in the neoadjuvant chemotherapy group were 79.8% and 68.0%, respectively, in contrast to the counterparts in the adjuvant chemotherapy group (89.5% each).

**Table 2:** Exposure to TS-1 for the scheduled study period of 365 days

	Neoadjuvant chemotherapy (*N* = 44)		Adjuvant chemotherapy (*N* = 19)	
	No.	%	No.	%
Planned schedules and doses	15	34.1	8	42.1
Mean relative dose intensity*	38	69.6	17	77.8
Dose reductions	6	13.6	5	31.6
Dose delays	12	27.3	7	36.8
Dose interruptions	20	45.5	3	15.8

Overall relative dose intensity, except 8 patients in the TS-1 group who relapsed: 72.3%

^*:^The mean of the actual-to-scheduled doses of TS-1 for 365 days

### Sites of relapse and deaths

Relapse occurred in 21 patients (50%) and 3 patients (15.8%) in the neoadjuvant and adjuvant chemotherapy groups, respectively (Table 3). The main sites of relapse were the lungs, locoregional tissues, brain, and distal lymph nodes in the neoadjuvant chemotherapy group and were the pleura, lungs, bone, and liver in the adjuvant chemotherapy group. Nineteen patients (13.1%) and 3 patients (15.8%) died in the neoadjuvant and adjuvant chemotherapy groups, respectively. One case of primary gastric cancer occurred in the neoadjuvant group.

**Table 3:** Sites of relapse in the neoadjuvant and adjuvant chemotherapy groups

	Neoadjuvant therapy group (*N* = 44)		Adjuvant therapy group (*N* = 19)	
	Relapse	Percent	Relapse	Percent
Sites of relapse (n)	21		3	
Locoregional tissues	9	42.9	0	0.0
Lungs	11	52.4	1	33.3
Pleura (pleural effusion)	2	9.5	2	66.7
Brain	7	33.3	0	0.0
Distal lymph nodes	5	23.8	0	0.0
Bone	1	4.8	1	33.3
Liver	1	4.8	1	33.3
Mediastinum	1	4.8	0	0.0

### Safety

Table 4 summarizes AEs. All AEs were 1 to 3 in CTCAE grade, and any grade 4 AEs did not occur. The most common AEs were leucocyte count decreased (50.8%), total bilirubin decreased (44.4%), pigmentation (42.9%), neutrophil count decreased (38.1%), aspartate aminotransferase increased (36.5%), alkaline phosphatase increased (36.5%), and constipation (30.2%). Grade 2 leukocyte count decreased (31.7%) was most frequent among hematologic AEs, and grade 1 pigmentation (41.3%) among nonhematologic AEs.

**Table 4:** Adverse events

	CTCAE grades* (*N* = 63)				
	1	2	3	Total	
	*n*	*n*	*n*	*n*	Percent
Hematologic, n					
Leukocyte count decreased	10	20	2	32	50.8
Neutrophil count decreased	5	13	6	24	38.1
Hemoglobin decreased	11	6	1	18	28.6
Platelet count decreased	17		1	18	28.6
Nonhematologic, n					
Febrile neutropenia			4	4	6.3
Total bilirubin increased	14	13	1	28	44.4
Aspartate aminotransferase increased	20	3		23	36.5
Alanine aminotransferase increased	14	3		17	27.0
Alkaline phosphatase increased	20	3		23	36.5
Creatinine increased	1	1		2	3.2
Hypersensitivity	1			1	1.6
Stomatitis (medical examination)	8	3	1	12	19.0
Stomatitis (dysfunction)	7	3		10	15.9
Anorexia	3			3	4.8
Nausea	11	2		13	20.6
Vomiting	1	2		3	4.8
Diarrhea	13	4	2	19	30.2
Constipation	4	1		5	7.9
Rash/desquamation	5	2		7	11.1
Pigmentation	26	1		27	42.9
Dysgeusia	3	1		4	6.3
Nail change	6	2		8	12.7
Fatigue	4		1	5	7.9
Edema	2			2	3.2
Neuropathy	2	1	1	4	6.3
Subcutaneous abscess		1		1	1.6
Epistaxis	1			1	1.6
Hypotension	3			3	4.8
Hand-foot syndrome	1	2	1	4	6.3
Arthralgia		1		1	1.6
Joint pain		1		1	1.6
Lassitude	1			1	1.6
Lacrimation	2	1		3	4.8
Blurred vision	1			1	1.6
Abdominal pain	1			1	1.6

*According to the Japanese version of the National Cancer Institute Common Terminology Criteria for Adverse.

## Discussion

The present clinical study—the first feasibility study of TAT—provides the following facts of clinical relevance: 1) only patients with TNBC, who had undergone adjuvant/neoadjuvant chemotherapy, were investigated; 2) therapeutic sustainability as assessed with RDI was not inferior to those obtained in the previous clinical studies of TS-1 alone or in combination [8, 12, 17]; 3) a high proportion (88.7%) of patients with TNBC had a non-pCR at baseline and were at high risk for relapse despite having undergone SPT; and 4) a median follow-up exceeded 6 years.

Regarding adjuvant chemotherapy with a fluoropyrimidine for breast cancer, meta-analyses on treatment with UFT (tegafur plus uracil) revealed that the drug was effective for breast cancer of stages I to IIIA [20] and for node-negative breast cancer [21], reducing the risk for relapse [20, 21]. In the present study, the 365-day cumulative rate of TS-1 administration was 68.3% that was comparable to 65.8% attained by the ACTS-GC Group-proposed chemotherapeutic regimen [9]—standard adjuvant chemotherapy for patients with advanced gastric cancer. Moreover, the mean RDI was 72.3% despite 88.7% of patients had a non-pCR. We found that the RFS rates (88.5%, 75.5%, and 68.6% at 1, 2, and 3 years of follow-up, respectively) and the OS rates (100.0%, 88.0%, and 82.8% at 1, 2, and 3 years of follow-up) in the adjuvant chemotherapy group were well maintained over 3 to 5 years after TS-1 administration, with significant differences against the counterparts in the neoadjuvant chemotherapy group at 5 years of follow-up. Furthermore, we did not find any large differences in the safety profile of TS-1 as compared with those in the phase II to III or feasibility studies of TS-1 alone or in combination [6–8, 11–14, 16, 17]. Hematologic AEs were predominant during the initial phase of TAT, and hepatic dysfunction, gastrointestinal symptoms, and skin manifestations occurred along with cumulative dose exposure. All AEs were manageable clinically, although grade 3 neutrophil count decreased occurred. Any grade 4 AEs did not develop. TAT was not severely affected by AEs, which confirmed the safety of TAT in patients with TNBC who relapsed after adjuvant/neoadjuvant chemotherapy. This dosing schedule of TS-1 was well tolerated by patients.

Recently, Shigekawa et al. [22] conducted a feasibility study of TS-1 in Japanese patients who had ER/PgR- or HER2-positive/negative breast cancer. They reported that the 365-day cumulative percentage of TS-1 administration was 66.4% and the percentage of eligible patients who completed the 18-course treatment was 51.2%; these values were equivalent to those reported by the ACTS-GC Group in Japanese patients with locally advanced gastric cancer [9]. They reported that grade 3 AEs were manageable and any grade 4 AEs did not occur. The hematologic and nonhematologic AEs of TS-1, which were assessed to be grade ≥ 3, were similar between their data and ours. Together, the feasibility of TAT, consisting of 14-day consecutive administration followed by 7-day drug holiday, was confirmed for the 365-day chemotherapeutic regimen of TS-1 (80 mg/m^2^ of BSA/day, BID, PO) in patients with MBC. The Japan Breast Cancer Research Group conducted CREATE-X, a phase III clinical study of postoperative adjuvant chemotherapy with or without capecitabine (control) in patients who had a non-pCR after surgery and neoadjuvant chemotherapy of the HER2-negative primary breast cancer [23]. The median age of patients in CREATE-X was 48 years (range, 25 to 74), and the study included 32.2% of patients who had TNBC. The subgroup analysis on patients with TNBC revealed 42% and 48% reductions in relapse (95% CI, 0.39–0.87) and OS (95% CI, 0.30–0.90) at 5 years in favor of the capecitabine group that showed the 5-year rates of DFS (69.8%) and OS (78.8%) rates as compared with the control group (56.1% and 70.3%, respectively). Namely, the 5-year RFS rate in the capecitabine group of CREATE-X was higher than the 5-year DFS rate in the neoadjuvant chemotherapy group of the present study. With respect to the safety profile of TS-1, AEs in the present study were similar in event type and exhibited differences in incidence as compared with those reported in previous clinical studies. However, the incidence of the hand-foot syndrome—the most common AE of capecitabine—was low (6.3%) in the present study compared to 73.4% in CREATE-X.

## Conclusions

Despite the fact that patients with TNBC had undergone adjuvant/neoadjuvant chemotherapy with an anthracycline and/or a taxane and were at high risk for relapse because of having a non-pCR or axillary lymph node metastases, TAT showed the completion rate that was comparable to those of the TS-1 studies in Japanese patients with gastric cancer or ER/PgR- and HER2-positive/negative breast caner. Additionally, TAT showed the acceptable profiles of efficacy and safety as compared with the previous clinical studies of TS-1 alone or in combination. Therefore, we conclude that TAT is potentially feasible as a novel posttreatment for patients with TNBC who underwent SPT.

## Data Availability

The raw data generated and analyzed during this study are not publicly available due to appropriate protection of patient personal information but are available from the corresponding author on reasonable request.
